# IL11 activates the placental inflammasome to drive preeclampsia

**DOI:** 10.3389/fimmu.2023.1175926

**Published:** 2023-05-24

**Authors:** Ellen Menkhorst, Leilani L. Santos, Wei Zhou, Guannan Yang, Amy L. Winship, Katarzyna E. Rainczuk, Philana Nguyen, Jian-Guo Zhang, Paddy Moore, Michelle Williams, Kim-Anh Lê Cao, Ashley Mansell, Evdokia Dimitriadis

**Affiliations:** ^1^ Department of Obstetrics and Gynaecology, The University of Melbourne, Parkville, VIC, Australia; ^2^ Gynaecology Research Centre, Royal Women’s Hospital, Parkville, VIC, Australia; ^3^ Centre for Reproductive Health, Hudson Institute of Medical Research, Clayton, VIC, Australia; ^4^ Department of Mathematics and Statistics, The University of Melbourne, Parkville, VIC, Australia; ^5^ Department of Anatomy and Developmental Biology, Development and Stem Cells Program, Biomedicine Discovery Institute, Monash University, Clayton, VIC, Australia; ^6^ Walter and Eliza Hall Institute, Parkville, VIC, Australia; ^7^ Department of Medical Biology, The University of Melbourne, Parkville, VIC, Australia; ^8^ Abortion and Contraception, Royal Women’s Hospital, Parkville, VIC, Australia; ^9^ Biomedical Animal Facility, The University of Melbourne, Parkville, VIC, Australia; ^10^ Centre for Innate Immunity and Infectious Diseases, Hudson Institute of Medical Research, Clayton, VIC, Australia; ^11^ Department of Anatomy and Developmental Biology, Monash University, Clayton, VIC, Australia

**Keywords:** interleukin 11, preeclampsia, ASC, NLRP3, inflammasome, hypertension, fibrosis

## Abstract

**Introduction:**

Preeclampsia is a life-threatening disorder of pregnancy unique to humans. Interleukin (IL)11 is elevated in serum from pregnancies that subsequently develop early-onset preeclampsia and pharmacological elevation of IL11 in pregnant mice causes the development of early-onset preeclampsia-like features (hypertension, proteinuria, and fetal growth restriction). However, the mechanism by which IL11 drives preeclampsia is unknown.

**Method:**

Pregnant mice were administered PEGylated (PEG)IL11 or control (PEG) from embryonic day (E)10-16 and the effect on inflammasome activation, systolic blood pressure (during gestation and at 50/90 days post-natal), placental development, and fetal/post-natal pup growth measured. RNAseq analysis was performed on E13 placenta. Human 1^st^ trimester placental villi were treated with IL11 and the effect on inflammasome activation and pyroptosis identified by immunohistochemistry and ELISA.

**Result:**

PEGIL11 activated the placental inflammasome causing inflammation, fibrosis, and acute and chronic hypertension in wild-type mice. Global and placental-specific loss of the inflammasome adaptor protein Asc and global loss of the Nlrp3 sensor protein prevented PEGIL11-induced fibrosis and hypertension in mice but did not prevent PEGIL11-induced fetal growth restriction or stillbirths. RNA-sequencing and histology identified that PEGIL11 inhibited trophoblast differentiation towards spongiotrophoblast and syncytiotrophoblast lineages in mice and extravillous trophoblast lineages in human placental villi.

**Discussion:**

Inhibition of ASC/NLRP3 inflammasome activity could prevent IL11-induced inflammation and fibrosis in various disease states including preeclampsia.

## Introduction

Preeclampsia is a life-threatening disorder of pregnacy unique to humans (1). Preeclampsia is a complex multi-system disease, diagnosed by sudden onset hypertension (>20 weeks gestation) and at least one other associated complication including proteinuria or placental or other maternal organ dysfunction (1, 2). Worldwide, over 4 million women develop preeclampsia each year (4.6% of pregnancies) (3) resulting in the death of 76,000 women and 500,000 babies (4, 5). Surviving mothers and babies have increased risk of future cardiovascular disease (6–8).

Poor implantation and placentation in the first trimester of pregnancy are widely accepted to be the sentinel causes of pregnancy diseases including early-onset preeclampsia (delivery <34 weeks gestation) (1, 9, 10). During early placentation, cytotrophoblast within the placental villus differentiate into syncytiotrophoblast and extravillous cytotrophoblast (EVT). The multinucleated syncytiotrophoblast lines the outer surface of the placental villus and is in direct contact with maternal blood, facilitating nutrient and gas exchange (9). EVT invade from anchoring placental villus into the uterine decidua and myometrium, replacing endothelial cells within uterine spiral arterioles to create low resistance vessels. Early-onset preeclampsia in particular is associated with inadequate EVT invasion and spiral artery remodeling, resulting in reduced uterine blood flow leading to placental ischemia (1). This placental dysfunction causes syncytiotrophoblast stress including endoplasmic reticulum stress, oxidative stress, and apoptosis (1). The stressed syncytiotrophoblast abnormally releases factors including anti-angiogenic agents and pro-inflammatory cytokines into maternal blood, causing systemic inflammation and widespread maternal endothelial dysfunction, resulting in the maternal syndrome of early-onset preeclampsia (1, 9, 11).

Interleukin (IL)11 is an IL6-type pleiotropic cytokine (12, 13). Despite IL11 inducing fibrosis in multiple tissues (14–17), the precise mechanism by which IL11 drives fibrosis is not understood. IL11 is elevated in maternal serum, placenta, and decidua from pregnancies with early-onset preeclampsia (15, 18). Demonstrating a causal role for IL11 in the etiology of early-onset preeclampsia, we have shown that IL11 inhibits human 1^st^ trimester primary trophoblast cell outgrowth (15) and invasion (19). Moreover, exogenous IL11 administration to pregnant mice during mid-gestation recapitulates preeclampsia-like features: hypertension, proteinuria, fetal growth restriction, and pre-term birth (15, 20). However, the precise mechanism by which IL11 causes placental damage and induces preeclampsia is unknown, although IL11 activates many pathways known to be altered in preeclampsia (15).

Inflammasomes are critical components of the inflammatory response implicated in the initiation of chronic low-grade inflammation (21). Inflammasomes are intracellular protein oligomers that sense pathogens, tissue injuries, and altered cellular homeostasis. Inflammasomes comprise a specific sensor protein (eg. NOD- LRR- and pyrin domain containing [NLRP]3), an adaptor protein (eg. PYD and CARD Domain Containing [PYCARD], hereafter referred to as ASC [Apoptosis-associated speck-like protein containing a CARD]) and an effector protein (eg. caspase-1). ASC is a key adaptor protein associated with many different sensor proteins, including NLRP3. Upon activation, the inflammasome forms an enzymatic complex that catalytically matures pro-IL1β and pro-IL18 into their respective bioactive forms and cleaves gasdermin D (GSDMD), releasing the N-terminal domain (GSDMD^NT^). This results in the formation of membrane pores, enabling the extracellular release of intracellular factors including IL1β, IL18, and High Mobility Group Box (HMGB)1 and causing cell death via pyroptosis. Whether IL11 activates inflammasomes has never been investigated, but we have previously shown that IL11 primes the decidual inflammasome in mice, up-regulating *Asc*, *Caspase-1*, and *Il1β* mRNA (20).

There is emerging evidence for elevated placental inflammasome activation in early-onset preeclampsia (1, 22–24); thus, we hypothesized that IL11 would activate placental inflammasomes resulting in cleaved IL1β, pyroptosis, and initiation of the cascade of events leading to preeclampsia. We aimed to determine the effects of IL11 on inflammasome activation in human and mouse placenta and whether loss of NLRP3/ASC-inflammasome activity could prevent IL11-induced preeclampsia.

## Methods

### IL11 treatment to induce preeclampsia *in vivo*

#### *In vivo* mouse experiments

All procedures were approved by the Animal Ethics Committees at Monash Medical Centre (B) (#MMCB-2017-27) and Melbourne University (#1814666). This study followed the NHMRC Australian Code of Practice for the Care and Use of Animals for Scientific Purposes. C57BL6 (wild-type, WEHI, Kew, VIC, Australia), *Asc*-/- (kindly provided by Millennium Pharmaceuticals), and *Nlrp3*-/- (kindly provided by the University of Lausanne) female (8-40 weeks; virgin 8-12 weeks at mating) and male (8-52 weeks) mice were housed under conventional conditions, had ad libitum food and water, and were maintained in a 12h light-dark cycle.

Mice were haphazardly assigned to experimental groups. There was no allocation concealment or blinding of the experimenter (required to give daily injections), however for the primary outcome (hypertension) the actual blood pressure was calculated by computer program and experimenters were blinded to outcome for histological assessments, qPCR, and Western blotting.

Pregnant mice were obtained using two methods: Single genotype pregnancy (wild-type, *Asc*-/- and *Nlrp3*-/-) where the day of vaginal plug detection was termed embryonic day (E) 0; and Embryo transfer pregnancy where *Asc*-/- embryos were implanted into wild-type recipient females to obtain pregnancies where only the placenta and fetus were *Asc*-/- (placental/fetal-specific *Asc-/-*). *Asc*-/- embryos were obtained by superovulation of *Asc*-/- (donor) females and subsequent mating with *Asc*-/- males. For superovulation, *Asc*-/- donor mice received 7.5IU of Pregnant Mare Serum Gonadotropin by subcutaneous injection on Day 1, followed by 7.5IU Human Chorionic Gonadotropin by subcutaneous injection on Day 3. Donor and recipient female mice were paired with a male on Day 3, plugged donor females were killed by cervical dislocation on Day 6 (equivalent to E2), and eight cell stage embryos were collected from oviducts before being transferred to the uterus of alert, restrained recipient females by non-surgical embryo transfer (25).

To determine the effect of elevated IL11 on placental development and pregnancy outcome, recombinant human IL11 (kind donation from Genetics Institute, TCO411) was PEGylated as described previously (15). Both mated females and females that received embryo transfer received once daily sub-cutaneous injections of 500µg/kg/day PEGIL11 or PEG control as previously published (endotoxin levels <2.3EU/ml), except injections were given from embryonic day (E)10 (plug detection, E0) to E16 (previously E17) (15). Pregnant mice were killed on E13 (n=4-6/group, wild-type and *Asc*-/-), E17 (n=6/group, *Asc*-/- only), or allowed to pup and dams kept alive until post-natal day (PN) 90 (n=5-6/group, wild-type, *Asc-/-*, *Nlrp3-/-* and placental/fetal-specific *Asc*-/-).

To determine the effect of a single dose of IL11 on placental gene expression, pregnant female wild-type mice at E13 were administered a single dose of 250µg/kg IL11 (non-PEGylated; endotoxin levels <0.1EU/ml) and implantation sites collected after 2h.

#### Serum and tissue collection

Mice were killed by tail vein injection of ketamine (235mg/kg) and xylazine (23.5mg/kg) followed by cardiac puncture to collect peripheral blood. After 2h incubation at room temperature, serum was separated from total blood by centrifugation at 500x*g* and snap frozen. Implantation sites (≥5/mouse) were dissected to obtain decidua/metrial lymphoid aggregate of pregnancy (MLAp), placenta, and fetus. The placenta/decidua/MLAp were weighed as a single unit and fixed in 10% neutral buffered formalin (NBF) or separated between the placenta and decidua/MLAp compartments and snap frozen on dry ice. Kidneys were snap frozen on dry ice or fixed for histology. The fetus and spleen were weighed. For statistical analyses data from ≥3-≤5 implantation sites/fetuses were averaged per dam.

#### Blood pressure measurements

Systolic Blood Pressure (sBP) was measured in conscious pregnant mice every 2-3 days from E9 to E17, PN50 to PN60, and PN90 to PN100 by tail-cuff plethysmography, following the procedure in the manufacturer’s manual (IITC Life Science and Kent Scientific) (26). Following 10 min stabilization on a preheated mat (25-30°C), 15 (5 acclimatization, 10 regular) consecutive automated inflation−deflation cycles were performed, and sBP calculated from by the software (Kent Scientific). Training prior to mating was not performed as pilot studies showed no benefit from this training, most likely as this occurred up to 3 weeks prior to the mouse becoming pregnant. Instead, mice were trained from E10-E12 in 2-3 sessions, before experimental readings were taken from E13 onwards. For postnatal readings, three measurements taken on different days between PN50-60 or PN90-100 were averaged.

### Human placental tissue isolation and culture

Human placental tissue was collected under Monash Health and Royal Women’s Hospital (#09317B) Human Research and Ethics Committee approval. Written and informed consent was obtained from each patient before surgery. Unless indicated, all reagents used for villus culture are sold as endotoxin-free/low.

#### Placental tissue collection

First- and second-trimester placental villus tissue was donated by healthy women undergoing pregnancy termination for psychosocial reasons (amenorrhea 6-24 weeks; n=73). Term placental villus and decidual tissue was donated by healthy women following spontaneous labor at term (>37 weeks; n=4).

#### Explant culture

First-trimester (6-12 weeks) placental villus was dissected and cultured in DMEM/F-12 (GIBCO) containing IL11 (100ng/ml; endotoxin levels <0.1EU/ml) or vehicle control (PBS) plus/minus the selective inhibitor of NLRP3, MCC950 (5µM; replaced every 24h; Sigma CP-456773 Sodium Salt; Sigma), or vehicle (H_2_O) under 5% CO_2_ in a humidified chamber for 22h or 72h. Explants were snap-frozen after 22h or fixed in 10% NBF after 72h. After 72h, conditioned media was centrifuged at 500*xg* to pellet cell debris before being snap frozen at -80°C.

#### Explant outgrowth assay

Dissected first-trimester villus (1mm x 1mm) was incubated overnight in serum-free media (DMEM/F12, no antibiotics) at 37°C and 5% CO2. The following morning, villus explants were placed onto a droplet (25µl) of Growth-Factor Reduced Matrigel™ (#356230, Corning) and allowed to adhere to the droplet for 3h at 37°C before being gently overlaid with serum-free media and cultured for 24h. Anchored explants were transfected with 10nM *ASC* siRNA (*PYCARD*, Ambion Silencer #4392420), or scramble control (Ambion Silencer #4390843) using Lipofectamine RNAiMax (Thermo Fisher Scientific, #13778150) according to the manufacturer’s instructions. After 24h of siRNA treatment, villus tips were photographed before IL11 (100ng/ml; endotoxin levels <0.1EU/ml) or vehicle (PBS) treatments were added. Explants were again photographed after 48h IL11 treatment and explant trophoblast outgrowth quantified using Image J (27). The outgrowth area was expressed as the percentage change in outgrowth following IL11 treatment. Representative explants were collected after 48h of siRNA treatment to confirm *ASC* knockdown by RT-qPCR.

### Gene expression

RNA was extracted from snap-frozen tissue and cells using the RNeasy Mini kit (QIAGEN) according to the manufacturer’s instructions. Genomic DNA was digested using the on column (RNase-free DNase set, #79256, QIAGEN) according to the manufacturer’s instructions. RNA concentration, yield, and purity were analyzed by spectrophotometry (Nanodrop Thermo Scientific, Scoresby, Victoria, Australia) at an absorbance ratio of A260/280nm.

#### RNA sequencing

The Illumina NovaSeq RNA-seq sequence production of a 100bp single-end run and the primary bioinformatics analysis was performed by the Australian Genome Research Facility (Project codes: CAGRF20114700, CAGRF21025523). After the RNA libraries were prepared using Illumina’s Ribo-zero Gold protocol, they were sequenced on an Illumina NovaSeq platform. Following primary bioinformatics analysis (demultiplexing and quality control) the data was processed through RNA-seq expression analysis workflow, including alignment, transcript assembly, quantification, and normalization. The cleaned sequence reads were then aligned against the *Mus musculus* genome (Build version mm10) using STAR aligner (v2.5.3a). mRNA were identified and kept according to MGI-Mouse Gene Expression Database (GXD) (28). Low raw count (lower than 15 in 13 samples or more) genes were removed. For each sample, counts were scaled and normalized by TMM (trimmed mean of M-values) and CPM (counts per million) using the R package edgeR (29). Differential expression analyses were performed using limma (Linear Models for Microarray) with empirical Bayes and voom procedure (30, 31). Heatmap of gene expression was generated by using the R package pheatmap. Genes were scaled on standard deviations by row and then clustered based on Pearson’s correlation using the complete-linkage method. Enrichment analysis was performed with the R package clusterProfiler (32) using over-representation analysis based on the hypergeometric test. The differential expressed genes were compared with the background gene lists from the Gene Ontology (GO) databases (33, 34). Cell composition deconvolution analysis used a series of bulk deconvolution methods benchmarked in (35), including CIBERSORT (source code kindly provided by The CIBERSORT Team) (36), OLS, FARDEEP (37), RLR (38), DCQ (39), elastic net, lasso, and ridge regression (40). Matrix were generated from the single-nuclei RNA-seq profile of cell type markers in (41): the pseudo-bulk expression profile of cell types were generated by the average expressions of the marker genes (35).The estimated cell proportions in each sample were then the average of results from above deconvolution methods. P-values were adjusted by the Benjamini-Hochberg procedure to control the false discovery rates under 0.05. All bioinformatics analyses were performed by R (4.1.0) on RStudio (1.3.1, RStudio, PBC).

#### RT-qPCR

RNA was reverse transcribed using Superscript III First-Strand Synthesis System (Thermo-Fisher) according to the manufacturer’s instructions, except 0.5µL Superscript III was included in each reaction. Real-time qPCR was performed using Power SYBR Green master mix (Applied Biosystems) on the Viia 7 fast block real-time qPCR system (Applied Biosystems) in triplicate (final reaction volume, 10μl) in 384-well Micro Optical plates (Applied Biosystems). A template-free negative control in the presence of primers and RNase-free water only negative controls were added for each run. Primer sequences are shown in **Table S1**; primers were obtained from Sigma-Aldrich. The qPCR protocol was as follows: 95°C for 10 min and 40 cycles of 95°C for 15s followed by 60°C for 1 min. Relative expression levels were calculated using comparative cycle threshold method (ΔΔCT) as outlined in the manufacturer’s user manual.

### Western blotting

Total protein was extracted from snap-frozen tissue by mechanical homogenization (QIAGEN Tissue Lyser; mouse tissues) or mortar and pestle (human villus) in Universal Lysis Buffer (50 mM Tris·HCl (pH 7.5), 150 mM NaCl, 2 mM EDTA, 2 mM EGTA, 25 mM NaF, 25 mM β-glycerophosphate, protease inhibitor mixture [Calbiochem]), before cell membranes were pelleted at 10,000x*g* and protein quantified (BCA assay, Pierce).

Denatured protein (40µg) was run on 4-15% SDS-PAGE gel (BioRad) for 1h at 150v before protein was transferred to PVDF or nitrocellulose membrane (TransBlot Turbo Transfer System, BioRad). Membranes were blocked (5% Skim Milk/TBS-0.1% Tween) for 1h before antibodies were incubated with primary antibodies (**Table S2**) overnight at 4°C. Membranes were washed then incubated with secondary antibody for 1h at RT followed by ECL (Clarity™ Western ECL Substrate, 5 min) and chemiluminescent bands visualized (iBright, BioRad).

### Histology and immunohistochemistry

Monash Histology Platform (Monash University) and Anatomical Pathology (Royal Children’s Hospital, Melbourne) processed and sectioned all formalin-fixed tissues and performed histological staining as required (Periodic Acid Schiff and Masson’s Trichrome).

Fresh mouse placenta/decidua and cultured human placental villus was fixed in 10% formalin at 4°C, washed 2x with TBS before processing and embedding in paraffin and sectioning at 5μm, before being placed onto SuperFrost slides, dried, deparaffinized, and rehydrated.

Dolichos Biflorus Agglutin (DBA) lectin (B-1035, Vector Laboratories) and isolectin B4 (ISB4; (#L2140 Sigma) staining were performed as per the manufacturer’s instructions to highlight decidual uNK cells and the extracellular matrix surrounding fetal blood vessels, respectively.

For immunohistochemical analysis, antigen retrieval (0.01M Sodium Citrate buffer, 5 min boiling) was followed by peroxidase activity block (3% hydrogen peroxide in methanol, 20 min at RT). Primary antibody (**Table S3**) or isotype negative control IgG were incubated 18h at 4°C. After stringent washing with 0.6% Tween-20 in TBS, antibody localization was detected by sequential application of biotinylated IgG (Goat anti-Rabbit Ig-biotin, 1:200, Vector Laboratories), avidin−biotin peroxidase complex (Vectastain Elite ABC kit, #PK-6100), and DAB (Dako). Sections were counterstained with hematoxylin and mounted.

#### Morphometry

Images were captured on an Olympus microscope (BX63) using an Olympus camera (DP80) and Cellsense Software.

##### Mouse placenta

Histology images are of mid-sagittal sections from hemisected implantation sites. All placental morphometry was quantified using Image J (27) or CellSense Software and performed on three separate fields of view. One placenta per mouse was analyzed and data collected from one section per placenta. To quantify collagen deposition and immunostaining for cleaved IL1β, cleaved caspase-1, GSDMD^NT^, or HMGB1, CellSense Software was used according to the manufacturer’s instructions. To assay vessel density, vessels stained with ISB4 in the middle region of the labyrinth were counted using Image J software. To assay glycogen cell area, Image J software was used to quantify the area of the junctional zone containing glycogen cells in PAS-stained sections. The sum of the total area for each field of view (3 fields of view quantified) gave the area of the junctional zone covered per placenta.

##### Mouse kidney

Collagen deposition in E13 kidneys was assessed by blue staining intensity (from 0, no staining to 3, intense staining). Collagen deposition around blood vessels and glomeruli was assessed separately by a blinded scorer.

##### Human

Placental immunostaining intensity was assessed (from 0, no staining to 3, intense staining) by two blinded scorers and the values from each scorer was averaged to give an intensity score.

### ELISA

#### FMS-like tyrosine kinase 1

Flt-1 concentration in mouse serum (diluted 1:5) was quantified by ELISA as per the manufacturer’s instructions (Mouse VEGFR1/Flt-1 Quantikine ELISA kit #MVR100 R&D systems).

#### Lactate dehydrogenase

Lactate dehydrogenase (LDH) release by placental villus explants in undiluted conditioned media was measured using the CyQUANT™ LDH Cytotoxicity Assay (C20300, Pierce) kit according to the manufacturer’s instructions.

### Statistics

Statistical analyses (excluding RNA sequencing analyses which are described above) were performed by GraphPad Prism version 9.2.0. *P*<0.05 was considered significant. Data were tested for normality and statistical tests (indicated in figure legends) chosen according to experimental design. A two-sided P value was calculated for all experiments.

## Results

### IL11 primed and activated the human placental villus inflammasome causing pyroptosis

**Figure 1:**
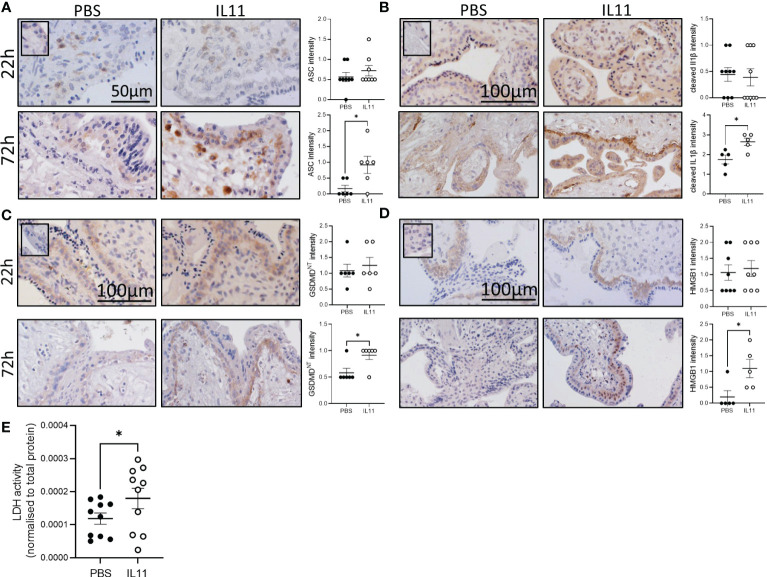
IL11 activated the placental inflammasome in human placental explants. **(A–D)** Immunostaining of ASC **(A)**, cleaved IL1β **(B)**, GSDMD^NT^ **(C)**, and HMGB1 **(D)** in human placental villus explants treated with IL11 (100ng/ml) for 22&72h. **(E)** LDH levels in conditioned media from human placental villus explants treated with IL11 (100ng/ml) for 72h. Data shows mean+SEM; Statistical tests: Mann-Whitney test **(A–D)**; Wilcoxon matched-pairs signed rank test **(E)**, *, p<0.05.

To determine whether IL11 primes or activates the human placental inflammasome, 1^st^ trimester placental villus explants were treated with IL11 for 22 and 72h (**Figures 1A–D**, **S1A**). IL11 treatment significantly increased placental villus *ASC* mRNA after 22h (**Figure S1A**), and villus stroma ASC immunostaining intensity after 72h (**Figure 1A**). Suggesting that IL11 may likewise induce ASC expression *in vivo*, mRNA production of *ASC* in placental villi collected across gestation mirrors placental villus *IL11* production; both increase during early gestation to peak at 14-16 weeks gestation before falling during the second trimester to low expression levels at term (**Figure S1B**). No other inflammasome component mRNA was primed by IL11 treatment (**Figure S1A**), however IL11 treatment of placental villus explants for 72h activated placental inflammasomes and caused villus pyroptosis. We saw significantly increased cytotrophoblast immunostaining for cleaved IL1β (**Figure 1B**), GSDMD^NT^ (**Figure 1C**), and HMGB1 (**Figure 1D**) and increased LDH release (**Figure 1E**) in IL11 treated villus.

In pregnant mice, a single injection of IL11 on E13 had no effect on any placental inflammasome-related gene expression after 2h (**Figure S1C**). Daily administration of PEGIL11 from E10-12 significantly up-regulated E13 placental *Il1β* and *Il18* mRNA expression (**Figure S1D**). There was no significant effect on placental Asc, Nlrp3, or pro-Il1β protein expression in the placenta (**Figure S2A**). We found a visual trend of stronger immunostaining for cleaved caspase-1, cleaved Il1β, and HMGB1 following PEGIL11 treatment, specifically in cells of villus trophoblast columns located within the placental labyrinth (42), but this was below the detection level of CellSense software (**Figures S2B–E**).

### Placental Asc is required for PEGIL11-induced preeclampsia features in mice

**Figure 2:**
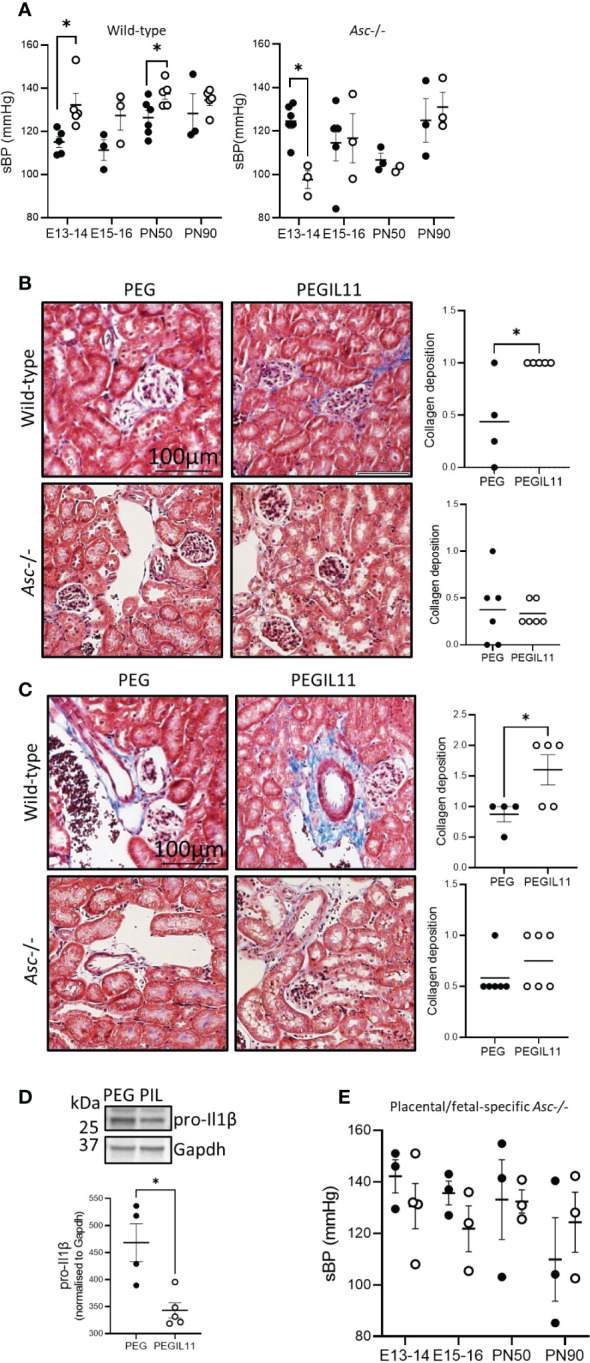
Loss of Asc-inflammasomes prevented development of PEGIL11-induced hypertension and fibrosis in pregnant mice. **(A)** Systolic blood pressure (sBP) in wild-type and *Asc*-/- mice. B-C. Masson’s trichrome stain collagen deposition (blue) around kidney glomeruli and tubules **(B)** and blood vessels **(C)** at E13 in wild-type and *Asc*-/- mice. **(D)** Immunoblot showing kidney pro-Il1β protein at E13 in wild-type mice after 3 days (E10-12) of treatment with PEGIL11. **(E)** Systolic blood pressure (sBP) in placental/fetal specific *Asc-/-* mice. ●, PEG treatment, ○, PEGIL11 treatment; PIL, PEGIL11; PN, post-natal day; Data shows mean+SEM; Statistical tests: Mann-Whitney test: **(A–E)**; *, p<0.05.

We previously showed that IL11 administration to pregnant mice induced features of preeclampsia including elevated blood pressure and impaired placentation (15). To confirm our previous results (15), we recapitulated this model of preeclampsia using PEGIL11. PEGIL11 treatment again significantly increased sBP in pregnant wild-type mice, although we found this occurred slightly earlier in gestation, at E13-14 (and was not sustained beyond E15, **Figure 2A**), whereas our previous study showed increased sBP at E16-17 (15). Excitingly, PEGIL11 administration during pregnancy in wild-type mice significantly increased sBP at PN 50 but this was lost at PN90 (**Figure 2A**). In the kidney, we previously found that IL11 treatment causes glomerular hypertrophy (15). Here we found kidney fibrosis at E13 in PEGIL11 wild-type treated mice, with significantly increased collagen deposition around glomeruli (**Figure 2B**) and renal blood vessels (**Figure 2C**). This corresponds to the gestational period in which we observed elevated systolic blood pressure in these mice (**Figure 2A**). We also saw reduced pro-Il1β protein in the kidney of PEGIL11-treated wild-type mice (**Figure 2D**), suggesting PEGIL11 treatment resulted in cleaved Il1β and renal inflammasome activation.

Given that IL11 activates the human placental inflammasome, we investigated whether IL11 acts via inflammasomes to drive preeclampsia in mice. We found that *Asc*-/- mice did not develop the PEGIL11-induced systemic features of preeclampsia seen in wild-type mice. PEGIL11 treatment significantly reduced sBP at E13-14 in *Asc*-/- mice (**Figure 2A**) but had no effect at E15-16, PN50, or PN90 (**Figure 2A**), and there was no evidence for kidney fibrosis (**Figures 2B, C**) or cleavage of Il1β (**Figure S3A**). It has previously been reported that hyperactivation of STAT3 results in splenomegaly (43): here PEGIL11 treatment induced splenomegaly in both wild-type and *Asc*-/- mice (**Figure S3B**), suggesting IL11-induced splenomegaly occurs *via* inflammasome-independent mechanisms.

As preeclampsia is thought to be driven by placental-released factors, we investigated the specific impact of placental *Asc* action (as opposed to systemic action) in this mouse model of preeclampsia. We performed embryo transfers to generate wild-type mothers carrying *Asc*-/- placentas and fetuses (placental/fetal-specific *Asc*-/-). The absence of placental and fetal *Asc* activity was sufficient to prevent PEGIL11-induced hypertension (**Figure 2E**), suggesting PEGIL11 acts via placental *Asc* to drive preeclampsia.

**Figure 3:**
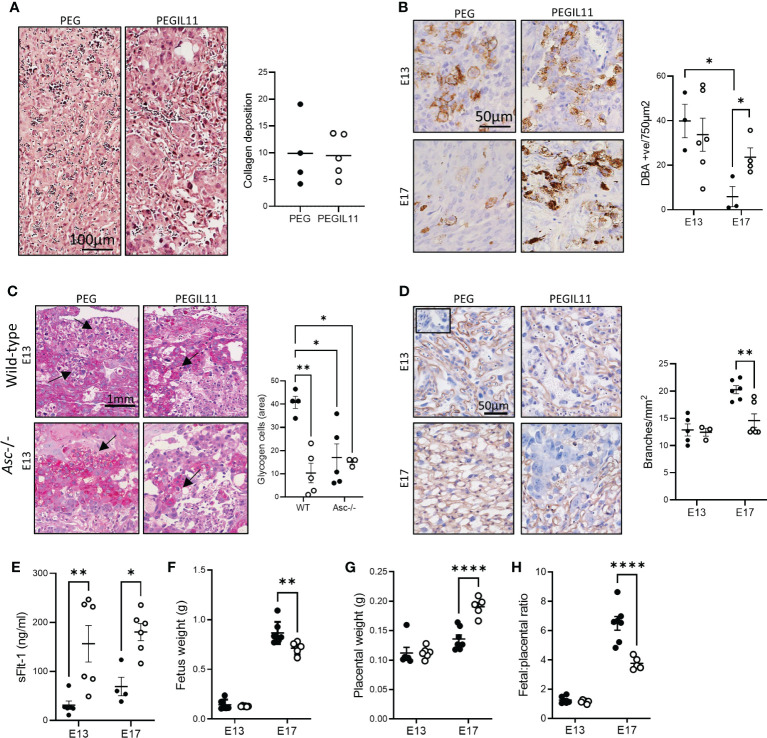
Loss of Asc-inflammasomes prevented some, but not all, PEGIL11-induced placental damage. **(A)** Masson’s trichrome stain showing no collagen deposition (blue) in E13 placental labyrinth of *Asc*-/- mice. **(B)** DBA positive cells (uterine Natural Killer cells) in *Asc*-/- E13 and E17 decidua. **(C)** PAS-stained junctional zone showing glycogen cells (arrows) in wild-type and *Asc*-/- mice. **(D)** ISB4 stained labyrinth zone identifying fetal vascular branching at E13 and E17 in *Asc*-/- mice; insert shows negative control. **(E)** Circulating sFlt-1 at E13 and E17 in *Asc*-/- mice. **(F)** Fetal weight in *Asc*-/- mice. **(G)** placental weight in *Asc*-/- mice, **(H)** fetal:placental ratio in *Asc*-/- mice. ●, PEG treatment; ○, PEGIL11 treatment; E, Embryonic Day; Data shows mean+SEM; Statistical tests: Mann-Whitney test: **(A, B)**; Two-way ANOVA: **(C–H)**; *, p<0.05; ** p<0.01; ****, p<0.0001.

To ascertain how PEGIL11-induced inflammasome activation affects placental formation, we compared the effect of PEGIL11 treatment between wild-type and *Asc*-/- mice. Placental collagen deposition, indicating placental fibrosis, is found in the labyrinth zone of IL11 treated wild-type mice at E17 (15) but this was absent in PEGIL11 treated *Asc*-/- mice (**Figure 3A**). Decidual uNK cell number at E13, previously decreased by IL11 treatment (15), was not changed by PEGIL11 treatment in *Asc*-/- mice. In control *Asc*-/- mice, uNK cell number significantly decreased from E13 to E17 which was not seen in PEGIL11-treated mice (**Figure 3B**). Glycogen cells, which store glycogen to provide a source of energy at term (44), were significantly reduced at E13 by PEGIL11 treatment in wild-type mice but not *Asc*-/- mice (**Figure 3C**). Interestingly, loss of *Asc*-/- itself was associated with a significant reduction in glycogen cell number, such that PEGIL11 treatment in *Asc*-/- mice had no further effect (**Figure 3C**). There was no effect of PEGIL11 treatment on cleaved caspase-1, cleaved Il1β, GSDMD^NT^, or HMGB1 immunostaining in *Asc*-/- mice (**Figures S3C–F**).

Surprisingly, despite our data showing that loss of *Asc* could ameliorate systemic features of PEGIL11-induced preeclampsia (**Figure 2**), we found that loss of *Asc* did not ameliorate all placental damage caused by PEGIL11 (**Figures 3D–H**). Matching our previously published data in wild-type mice (15), we found that in *Asc*-/- mice PEGIL11 treatment significantly impaired placental labyrinth zone vascular branching at E17 (**Figure 3D**) and increased circulating (serum) sFlt-1 at both E13 and 17 (**Figure 3E**). Correspondingly, loss of *Asc* did not prevent fetal growth restriction at E17 (**Figure 3F**), which was associated with increased placental weight at E17 (**Figure 3G**) and a decreased fetal:placental ratio (**Figure 3H**).

### PEGIL11 increased placental cathepsin production to drive preeclampsia features

**Figure 4:**
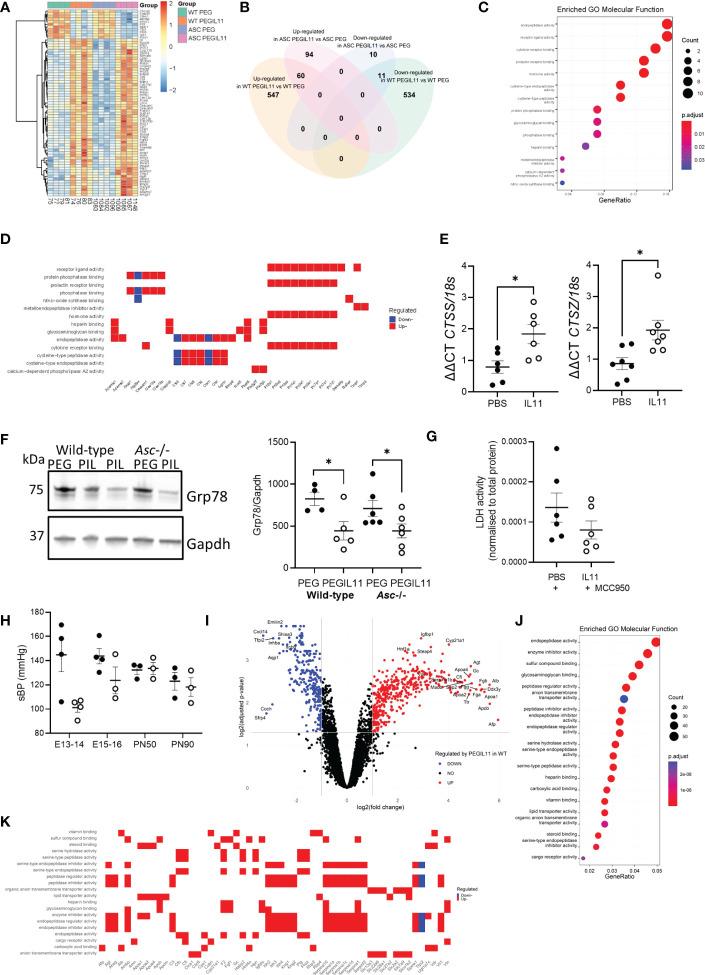
Differentially expressed protein-coding genes identified by RNAseq in both wild-type and *Asc*-/- placenta following PEGIL11 treatment. **(A)** Heatmap of differentially expressed genes. **(B)** Venn diagram of differentially expressed genes in the two genetic models. **(C)** Enriched GO Molecular Functions identified by analysis of differentially expressed genes found in both wild-type and *Asc*-/- placenta following PEGIL11 treatment. **(D)** Molecular function of differentially expressed genes following PEGIL11 treatment in both wild-type and *Asc*-/- placenta. **(E)** *CTSS* and *CTSZ* mRNA expression in human placental villus explants treated with IL11 (100ng/ml) for 22h. **(F)** Placental ER molecular chaperone Grp78 protein expression in placenta from wild-type and *Asc*-/- mice. **(G)** LDH levels in conditioned media from human placental villus explants treated with PBS/IL11 (100ng/ml) + MCC950 (5µM) for 72h. **(H)** systolic blood pressure (sBP) in *Nlrp3*-/- mice treated with PEGIL11. **(I)** Volcano plot of differentially expressed genes identified in wild-type but not *Asc*-/- mice following PEGIL11 treatment. **(J)** Enriched GO Molecular Functions identified by analysis of differentially expressed genes found in only wild-type placenta following PEGIL11 treatment. **(K)** Molecular function of differentially expressed genes (with logFC >2.5 or < -2.5 and adjusted p-value <0.005 for clear visualization) identified in only wild-type placenta. ●, PBS/PEG treatment; ○, IL11/PEGIL11 treatment; E, Embryonic Day; PIL, PEGIL11; PN, Post-natal Day; Data shows mean+SEM; Statistical tests: Empirical Bayes moderated t-test: A, Wilcoxon matched-pairs signed rank test: E, G; one-way ANOVA: F; Mann-Whitney t-test: H; Hypergeometric test: I; *, p<0.05.

To understand how PEGIL11 alters placentation and causes inflammasome activation and hypertension in mice, we performed RNA sequencing on E13 placental tissue from PEGIL11-treated wild-type and *Asc*-/- mice (**Figures 4**; **S4**). PEGIL11 treatment significantly altered the expression of 1,152 genes in wild-type mice (**Figures S4A–C**) and 175 in *Asc-/-* mice (**Figures S4D–F**). To define the PEGIL11 regulated factors that may activate the inflammasome, we identified 71 genes which were significantly regulated by PEGIL11 in both wild-type and *Asc*-/- mice (**Figures 4A, B**, **S4G–I**). All these genes showed regulation in the same direction (up- or down-regulation) by PEGIL11 in both wild-type and *Asc-/-* mice. The most significant enriched molecular functions regulated by PEGIL11 in both wild-type and *Asc-*/- mice were associated with receptor binding, specifically cytokine and prolactin receptor binding and endopeptidase activity (**Figure 4C**), including regulation of multiple cathepsins (*Cts*), with 4 upregulated and 2 downregulated cathepsin family genes (**Figure 4D**). Cathepsins are well known to activate the NLRP3 inflammasome (45), therefore we investigated whether IL11 likewise regulates cathepsin production and NRLP3 inflammasome activation in human placenta. IL11 treatment significantly increased human placental villus mRNA expression of cathepsin S (*CTSS*) and Z (*CTSZ*) (**Figures 4E**, **S5A**). ER stress is another well characterized mechanism that causes NLRP3 activation (22, 46). Therefore, we investigated whether PEGIL11 regulated production of ER chaperone proteins in wild-type and *Asc*-/- placentas. PEGIL11 treatment significantly reduced placental 78 kDa glucose-regulated protein (Grp78, also known as Binding immunoglobulin protein, BiP) protein in wild-type and *Asc*-/- mice (**Figure 4F**) but had no effect on endoplasmic reticulum protein 44 (Erp44) or Glucose-regulated protein 94 (Grp94) (**Figure S5B**).

To confirm that IL11 activated NLRP3 inflammasomes, we treated human placental villus explants with IL11 plus the NLRP3 inhibitor, MCC950. Co-treatment with MCC950 prevented the IL11-induced LDH production (**Figure 4G**) seen previously (**Figure 1E**), indicating that IL11 likely caused placental pyroptosis (47) via activation of the NLRP3 inflammasome. Further, no elevation in systolic blood pressure following PEGIL11 treatment was seen in *Nlrp3*-/- mice during gestation or at PN50 or PN90 (**Figure 4H**).

As our *in vivo* data demonstrated that activation of the placental inflammasome is required to induce hypertension in our mouse model of preeclampsia, we investigated whether IL11 regulated expression of genes likely to drive hypertension via inflammasome activation. To do this we identified genes which were regulated by PEGIL11 treatment in wild-type mice but not *Asc-/-* mice (**Figure 4I**). The most significant enriched molecular functions of genes regulated only in wild-type mice were associated with endopeptidase activity, enzyme inhibitor activity, and sulfur compound activity (**Figure 4J**). We identified that PEGIL11 treatment significantly regulated the placental production of multiple factors previously associated with preeclampsia including alpha-fetoprotein, angiotensinogen, apolipoproteins A1 and B, and vitamin D receptor (**Figure 4K**).

### PEGIL11-induced poor fetal outcomes were not resolved by loss of *Asc* or *Nlrp3*

**Figure 5:**
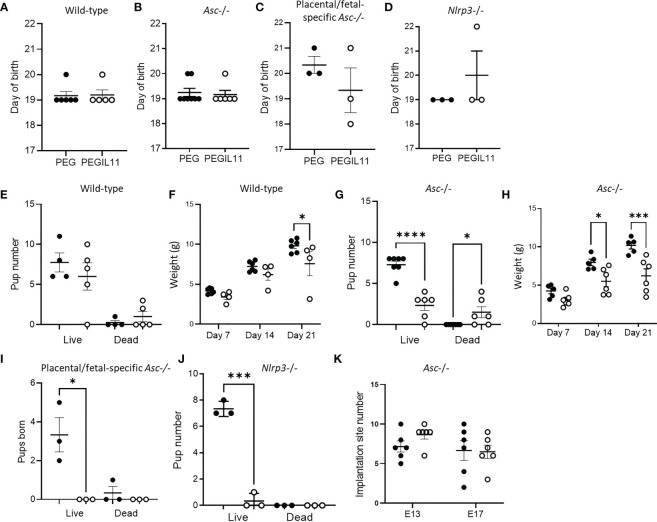
Loss of inflammasome activity does not resolve IL11 induced poor pregnancy outcomes. **(A–D)**. Day of birth in wild-type **(A)**, *Asc*-/- **(B)**, placental/fetal specific *Asc*-/- **(C)**, and *Nlrp3*-/- **(D)** mice. **(E)** Pup number on day of birth in wild-type mice. **(F)** Pup weight post-natal days 7, 14, and 21 in wild-type mice. **(G)** Pup number on day of birth in *Asc*-/- mice. **(H)** Pup weight post-natal days 7, 14, and 21 in *Asc*-/- mice. **(I)** Pup number on day of birth in placental/fetal specific *Asc*-/- mice. **(J)** Pup number on day of birth in *Nlrp3*-/- mice. **(K)** Implantation site number and pup number on day of birth in *Asc*-/- mice. ●, PEG treatment; ○, PEGIL11 treatment; E, Embryonic Day; Data shows mean+SEM; Statistical tests: Mann-Whitney: test **(A–E)**,; Kruskal-Wallis test: **(E, G, I–K)**; Repeated measures ANOVA: **(F, H)** *, p<0.05; ***, p<0.001; ****, p<0.0001.

In our previous study, IL11 treatment from E10-17 caused preterm birth, stillbirth and reduced postnatal growth. In this study, we ceased PEGIL11 injections at E16, and found no preterm birth in any genetic background (**Figures 5A–D**). In wild-type mice this was associated with prevention of stillbirth (**Figure 5E**) and restored postnatal growth (**Figure 5F**), however, in contrast, PEGIL11 treated *Asc*-/- dams had significantly fewer live-born pups (**Figure 5G**) and reduced postnatal growth (**Figure 5H**). PEGIL11 treatment of placental/fetal-specific *Asc*-/- (**Figure 5I**) and *Nlrp3*-/- mice (**Figure 5J**) resulted in almost no live births.

To investigate when PEGIL11 was causing fetal/pup loss in *Asc*-/- mice, we counted implantation site number at E13 and 17. Implantation site number was no different between the PEG and PEGIL11-treated animals on E13 or E17 (**Figure 5K**), but there were significantly fewer pups born alive to PEGIL11-treated *Asc*-/- mice (**Figure 5G**), suggesting PEGIL11 treatment caused late-gestation fetal loss or stillbirth in this genetic background.

### PEGIL11 impaired trophoblast differentiation via inflammasome-independent mechanisms

**Figure 6:**
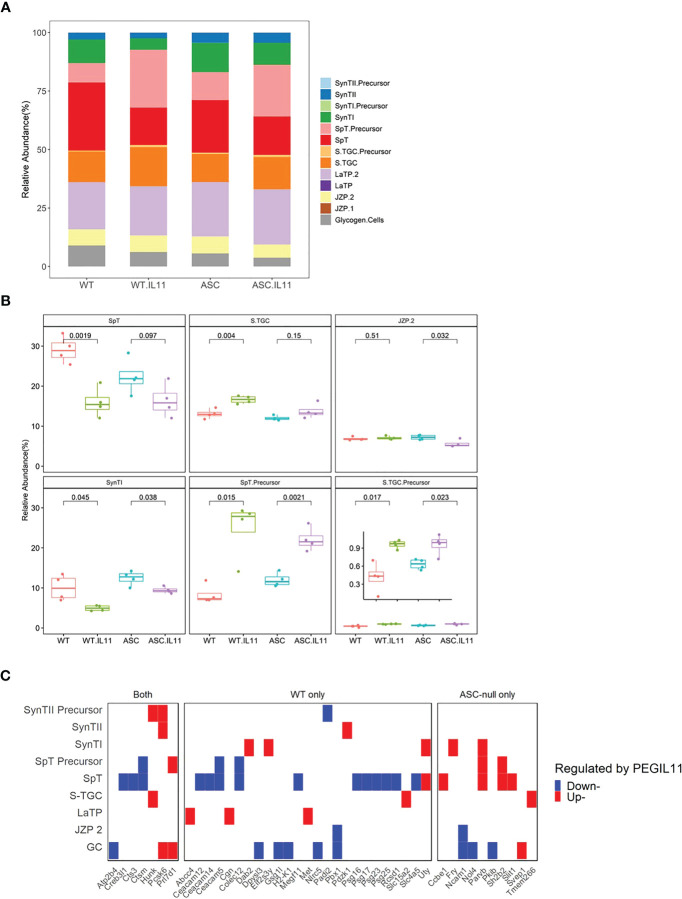
PEGIL11 impaired trophoblast differentiation and placental function in mice. **(A)** Estimated trophoblast cell lineages following PEGIL11 treatment in wild-type and *Asc*-/- placenta at Embryonic day (E)13. **(B)** Boxplots of estimated trophoblast cell lineages regulated by PEGIL11 treatment at E13 in wild-type and *Asc*-/- placenta. **(C)** Trophoblast cell lineage markers (fold-changes > 2) regulated by PEGIL11 treatment in wild-type and *Asc*-/- placenta. Data shows median (bold line), interquartile range (IQR, box), and range (bar); Statistical tests: student’s t-test. WT, wild-type; WT.IL11, PEGIL11 treatment in wild-type; ASC, *Asc-/-*; ASC.IL11, PEGIL11 treatment in *Asc-/-*. SpT, spongiotrophoblasts; S.TGC, Sinusoidal trophoblast giant cells; SynTI, syncytiotrophoblast layer 2 (the outermost syncytiotrophoblast layer); SynTII, syncytiotrophoblast layer 2; JZP2, Junctional zone precursors 2; LaTP, labyrinth trophoblast progenitor; GC, glycogen cells.

Prolactin (Prl)-family genes are well established markers of trophoblast lineage in mouse placenta. We identified that the Prl family was highly affected by PEGIL11 treatment in both wild-type and *Asc-/-* mice, with 9 upregulated Prl family genes in the E13 mouse placenta (**Figure 4D**). Using cell type deconvolution with single-nuclei RNA-seq expression profile of trophoblast subtypes (41) as reference, we found that PEGIL11 treatment was associated with significant changes in the relative proportion of trophoblast lineages in wild-type and *Asc-/-* mice (**Figures 6A, B**). Wild-type and *Asc-/-* mice both showed reduced Syncytiotrophoblast lineage I (SynTI) and increased precursors to spongiotrophoblast (SpT) and spongiotrophoblast giant cell (S.TGC) lineages; in wild-type mice alone we found decreased SpT lineage and increased S.TGC lineage; in *Asc-/-* mice only we found decreased junctional zone precursor (JZP) lineage 2 (JZP.2) (**Figures 6A, B**).

By comparing trophoblast cell subtype markers to the differential expressed genes following PEGIL11 treatment we found that a wide range of markers were decreased in wild-type mice but not *Asc-/-* (**Figures 6C**, **S5C**), implying potential absolute cell loss only in PEGIL11-treated wild-type mice.

**Figure 7:**
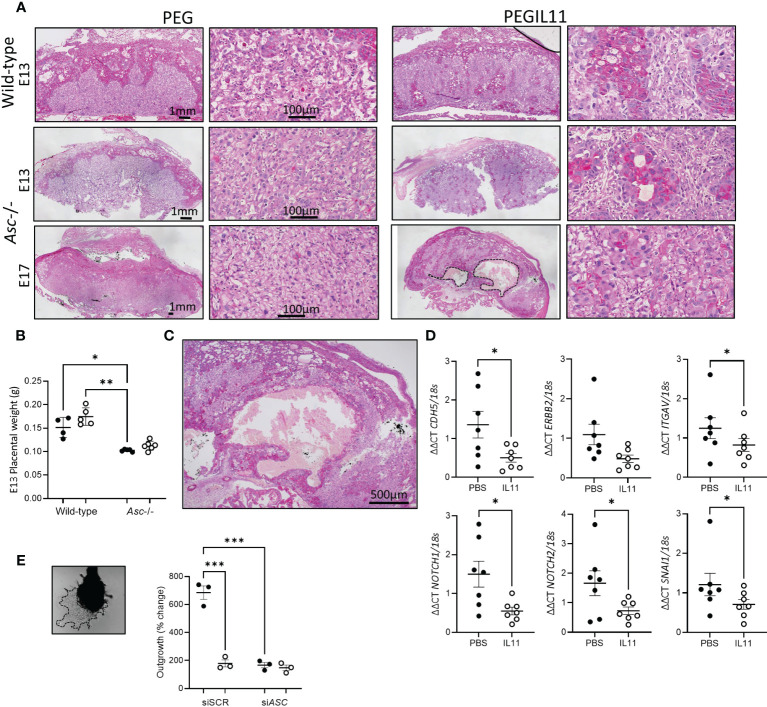
**(A)**. PAS-stained mouse wild-type and *Asc*-/- placenta showing retained villus trophoblast in the labyrinth (higher power). Hemorrhage in Embryonic day **(E)** 17 PEGIL11 treated *Asc*-/- placenta outlined in a dashed line. Representative images, n=4-6/group. **(B)** Placental weight at E13 in wild-type and *Asc*-/- mice. **(C)** High power image of hemorrhage in **(A)**. **(D)**. *CDH5, ERBB2*, *ITGAV*, *NOTCH1*, *NOTCH2*, and *SNAI1* mRNA expression in human placental villus explants treated with IL11 (100ng/ml) for 22h. **(E)** Extravillous trophoblast outgrowth from placental villus tips. Dashed line shows area of trophoblast outgrowth from explant tip. SpT, spongiotrophoblast, SynTI, syncytiotrophoblast 1. ●, PEG/PBS treatment; ○, PEGIL11/IL11 treatment; Data shows mean+SEM; Statistical tests: one-way ANOVA: B, Wilcoxon matched-pairs signed rank test: D; Repeated measures ANOVA: E; *, p<0.05; ** p<0.01; ***, p<0.001.

Using histological analyses, we visualized altered trophoblast differentiation in wild-type and *Asc*-/- placenta following PEGIL11 treatment (**Figure 7**). Dense villus trophoblast columns (42) were visible in the labyrinth of PEGIL11-treated wild-type and *Asc*-/- mice (**Figure 7A**). At E13, placentas from *Asc*-/- mice weighed significantly less than placentas from wild-type mice (**Figure 7B**), however there was no significant effect of genetic background on fetal weight (**Figure S6A**) or the fetal:placental ratio (**Figure S6B**) at this stage of gestation. Areas of hemorrhage in the placental labyrinth were seen at E17 in 5/6 PEGIL11-treated *Asc*-/- mice (**Figure 7C**) but not in PEGIL11-treated wild-type treated mice (15).

IL11 likely also regulates trophoblast differentiation in human placental villus: IL11 treatment significantly inhibited placental villus explant expression of markers associated with the extravillous trophoblast linage (**Figures 7D**, **S6C, D**) (41) and impaired extravillous trophoblast outgrowth *in vitro* (**Figure 7E**), as previously published (15). Loss of *ASC* itself also significantly reduced trophoblast outgrowth compared to scramble control, equivalent to IL11 treatment (**Figures 7E**, **S6E**).

## Discussion

Here we present the first evidence that IL11 drives activation of the ASC/NLRP3 inflammasomes, causing villus pyroptosis, placental and renal fibrosis, and the maternal syndrome of preeclampsia, including chronic post-natal hypertension, in mice. We further showed that IL11 had inflammasome-independent actions resulting in dysregulated trophoblast differentiation, placental damage, and likely impaired placental function, leading to fetal growth restriction and perinatal death.

There is substantial evidence for placental inflammasome activation, particularly NLRP3 inflammasome activation, and pyroptosis in early-onset preeclampsia (22, 23). Inflammasome activation causes the release of the pro-inflammatory cytokines IL1β and IL18 which are known inducers of renal and vascular dysfunction (21). Placental villus explants release highly variable levels of IL1β (and IL18) under control conditions due to constitutive NLRP3 inflammasome activity – without detectable Gasdermin D cleavage (48), suggesting that constitutive placental inflammasome activity is critical for healthy placental function. However, preeclamptic placentas secrete higher levels of IL1β into the maternal circulation (49) and key components of the inflammasome including ASC, cleaved caspase-1, GSDMD^NT^, and HMGB1 are increased in the placenta from women with preeclampsia (22, 24, 50). Our *in vitro* data shows that IL11-induced activation of the inflammasome in the human placental villus results in Gasdermin D cleavage and pyroptotic cell death, thus placental exposure to excess IL11 may cause hyperactivation of the placental inflammasome, resulting in Gasdermin D cleavage, pyroptotic cell death, and placental damage leading to preeclampsia.

Although IL11 primes *ASC* in human placental villus, it is unlikely that IL11 directly activates the placental inflammasome: direct inflammasome activation is rapid, whereas here inflammasome activation markers were found in placental villus only at 72h after IL11 treatment. This suggests that IL11 stimulates the production of other factors which then activate the inflammasome. NLRP3 is the primary inflammasome associated with hypertension (21) and is activated by cathepsins (45) and ER stress (22, 46). NLRP3 activation and ER stress induces placental release of pregnancy-incompatible factors including sFlt-1, IL1β, and other pro-inflammatory cytokines and alarmins into maternal circulation causing oxidative stress, systemic inflammation, and endothelial dysfunction (22). Here we demonstrated that the expression of cathepsins S and Z is induced by IL11 in human placenta. Cathepsins S and Z are both known to activate the NLRP3 inflammasome in other tissues (45) and have previously been identified in trophoblast and placenta (51–53). We have previously shown that IL11 regulates the production of many ER factors (54, 55) and here we showed that PEGIL11 down-regulates Grp78 in mouse placenta. GRP78 is a critical regulator of the Unfolded Protein Response (UPR) and its loss drives ER stress (56, 57). Placental ER stress and the UPR are activated in early onset preeclampsia (58). Altogether this data suggests that elevated placental IL11 may trigger placental inflammasome activation via cathepsin activity and inducing ER stress.

Restricting *Asc* loss to the placenta and fetus was sufficient to protect against IL11-induced hypertension during pregnancy and at 50 days post-natal, indicating IL11 drives hypertension via placental-inflammasome activation in this mouse model. This is supported by our previous study where IL11 administration had no effect in non-pregnant mice (15). Interestingly, circulating sFlt-1, which is thought to contribute to endothelial dysfunction leading to hypertension and proteinuria in women (9), remained elevated in PEGIL11-treated *Asc*-/- mice. This phenomenon is observed in other murine models of preeclampsia (26, 59–61) where circulating sFlt-1 levels are not associated with the development or prevention of preeclampsia features. Moreover, a recent clinical trial showed that, while metformin treatment prolonged gestation in women with early-onset preeclampsia, it had no effect on circulating levels of anti-angiogenic markers including sFLT-1 (62). This suggests that, at least in mouse models of preeclampsia, sFlt-1 may reflect placental cellular stress rather than directly inducing hypertension or proteinuria (61, 63).

IL11 has an emerging role as an inducer of fibrosis in multiple tissues, including the placenta (15), lung (14), liver (17), heart (16), and kidney (16). Fibrosis is seen in the vessels and kidneys of hypertensive patients, in animal models of hypertension (21), and in the placenta of women with preeclampsia (64). The data presented here demonstrate for the first time that IL11 acts via the Asc-inflammasome to induce placental and renal fibrosis, with loss of *Asc* preventing IL11 induced fibrosis in the placenta and kidney. We also found that IL11 treatment increased ASC immunostaining in the stroma of the human placental villus, the location of fibrosis in preeclampsia (65).

This study clearly demonstrates that IL11 regulates trophoblast differentiation via Asc-dependent and independent pathways. Trophoblast differentiation was significantly altered by IL11 treatment in both wild-type and *Asc*-/- mice, resulting in the loss of markers associated with spongiotrophoblast and syncytiotrophoblast, retention of dense villus trophoblast columns in the labyrinth, and reduced labyrinth branching. Interestingly, endoplasmic reticulum (ER) stress is also known to disrupt trophoblast differentiation in mouse placentas (66–68), suggesting that IL11 may induce both NLRP3 inflammasome activation and impair trophoblast differentiation via its regulation of ER factors including Grp78.

Supporting a role for IL11 in human trophoblast differentiation, here we found that IL11 reduced the expression of the EVT markers cadherin 5 (CDH5), integrin subunit alpha V (ITGAV), neurogenic locus notch homolog protein (NOTCH) 1/2 and snail family transcriptional repressor 1 (SNAI1) in placental villus explants. Further, whilst IL11 promotes migration of already differentiated primary EVTs (69), IL11 inhibits EVT outgrowth from placental villus explants (15), likely due to a reduction in differentiation of cells towards the EVT lineage. Surprisingly, we saw IL11-induced inflammasome activation only in cytotrophoblast cells, with no immunostaining for activated inflammasome components detected in the syncytiotrophoblast. This may be an artifact of the explant culture method (70, 71) as we found PEGIL11 treatment reduced SynTI markers and also Grp78 *in vivo*, which interacts with α_2_-macroglobulin to promote trophoblast cell (BeWo) fusion (72).

Although cessation of PEGIL11 treatment at E16 prevented pre-term birth, fetal outcomes remained poor in *Asc*-/-, *Nlrp3-/-*, and placental/fetal specific *Asc-/-* mice. This is likely due to impaired trophoblast differentiation into the highly branched labyrinthine trophoblast which surround the maternal-origin vascular channels (42) and areas of placental hemorrhage compromising placental function, likely leading to the poor fetal outcomes seen in this study. Certainly, parenchymal infarcts are found in preterm stillbirth (73, 74). It is somewhat surprising that PEGIL11-treatment did not cause as significant placental damage in wild-type mice. We speculate that inflammasome activation, whilst causing systemic negative consequences in the form of preeclampsia symptoms, may protect the placenta by initiating fibrosis and tissue repair mechanisms.

In conclusion, here we demonstrate that IL11 activated the placental inflammasome, causing pyroptosis in human placental villus and the features of preeclampsia in a murine model. This is the first evidence that IL11 activates the inflammasome in any tissue. Therapeutic inhibition of the ASC or NLRP3 inflammasome in women with elevated serum IL11 in early pregnancy could prevent IL11-induced placental inflammation and subsequent preeclampsia. The development of therapeutics to block IL11-induced inflammation and fibrosis are the current focus in many disease states: this study suggests that therapeutically targeting the inflammasome could prevent IL11-induced inflammation and fibrosis in a variety of tissues, including the placenta. However, this study also indicates that simply inhibiting placental inflammasome activity to prevent preeclampsia-associated hypertension does not protect against placental insufficiency and inhibiting IL11 action is also required to improve fetal outcomes. This study has far-reaching implications on our understanding of the mechanisms by which IL11 causes inflammation and fibrosis.

## Data availability statement

The datasets presented in this study can be found in online repositories. The names of the repository/repositories and accession number(s) can be found below: https://www.ncbi.nlm.nih.gov/ GSE193685.

## Ethics statement

The studies involving human participants were reviewed and approved by Monash Health Human Research and Ethics Committee (#09317B) Royal Women’s Hospital Human Research and Ethics Committee (#09317B). The patients/participants provided their written informed consent to participate in this study. The animal study was reviewed and approved by Monash Medical Centre (B) (#MMCB-2017-27) Melbourne University (#1814666).

## Author contributions

EM, conceptualization, formal analysis, funding acquisition, investigation, methodology, writing – original draft, writing – review and editing; LS, investigation, methodology, writing – review and editing; WZ, investigation, writing – review and editing; GY, data curation, formal analysis, writing – original draft; AW, investigation, methodology; KR, investigation; PN, investigation; J-GZ, resources, PM, resources; MW, methodology, investigation; K-AC, methodology, supervision; AM, resources, conceptualization; ED, conceptualization, funding acquisition, methodology, project administration, supervision, writing – review and editing. All authors contributed to the article and approved the submitted version.
