# Supplementary material for: IL11 activates the placental inflammasome to drive preeclampsia
Source: Front Immunol. 2023 May 24;14:1175926. doi: 10.3389/fimmu.2023.1175926 (PMC10244672; doi:10.3389/fimmu.2023.1175926)
Supplement: Supplementary file 1 [file DataSheet_1.docx]

Supplementary Material

**IL11 activates the placental inflammasome to drive preeclampsia**

**Ellen Menkhorst, Leilani L Santos, Wei Zhou, Guannan Yang, Amy L Winship, Katarzyna E Rainczuk, Philana Nguyen, Jian-Guo Zhang, Paddy Moore, Michelle Williams, Kim-Anh Lê Cao, Ashley Mansell, Evdokia Dimitriadis*.**

*** Correspondence:** Evdokia Dimitriadis: evdokia.dimitriadis@unimelb.edu.au

**Supplementary Table 1** Primer sequences for human and mouse primers used.

|  | **Gene** | **Forward 3`-5`** | **Reverse 3`-5`** |
| --- | --- | --- | --- |
| ***Both*** | *18s* | GATCCATTGGAGGGCAAGTCT | CCAAGATCCAACTACGAGCTT |
| ***Human*** | *AIM2* | TCAAGCTGAAATGAGTCCTGC | CTTGGGTCTCAAACGTGAAGG |
|  | *ASC* | AGGCCTGCACTTTATAGAC | CTTCCCGTACAGAGCATC |
|  | *CASP1* | CAACTACAGAAGAGTTTGAGG | AACATTATCTGGTGTGGAAG |
|  | *CDH5* | TTGGAACCAGATGCACATTGAT | TCTTGCGACTCACGCTTGAC |
|  | *CTSB* | GAGCTGGTCAACTATGTCAACA | GCTCATGTCCACGTTGTAGAAGT |
|  | *CTSC* | CCAACTGCACCTATCTTGACC | AAGGCAAACCACTTGTAGTCATT |
|  | *CTSL1* | CTTTTGCCTGGGAATTGCCTC | CATCGCCTTCCACTTGGTC |
|  | *CTSL2* | CGTGACGCCAGTGAAGAATCA | CGCTCAGTGAGACAAGTTTCC |
|  | *CTSS* | AAACGGCTGGTTTGTGTGC | CAGTGGTGATCCAGGGTAGG |
|  | *CTSZ* | CAGCGGATCTGCCCAAGAG | CGATGACGTTCTGCACGGA |
|  | *ERBB2* | AGCCGCGAGCACCCAAGT | TTGGTGGGCAGGTAGGTGAGTT |
|  | *ERVW1* | CCCCATCGTATAGGAGTCTT | CCCCATCAGACATACCAGTT |
|  | *ERVFRD1* | AGCCTTAACGACCATGCAAGA | CTGTGCTGCCGTTAACATGTC |
|  | *GSDMD* | CATGGCATCGTAGAAGTGGAAG | CAGGCGGTGCCTATGTCTC |
|  | *hCGβ* | ATGGACTCGAAGCGCACATC | GCTACTGCCCCACCATGACC |
|  | *HMGB1* | AGAAGTGCTCAGAGAGGTGGA | CCTTTGGGAGGGATATAGGTT |
|  | *IL1β* | CAGCCAATCTTCATTGCTA | TCGGAGATTCGTAGCTGGAT |
|  | *IL11* | GTGGCCAGATACAGCTGTCGC | GGTAGGACAGTAGGTCCGCTC |
|  | *IL18* | GCATCAACTTTGTGGCAATG | TCCGGGGTGCATTATCTCTA |
|  | *ITGAV* | GAAAAGAATGACACGGTTGC | AGTGATGAGATGGTCCCCGCT |
|  | *ITGA6* | TGCTGTTGGTTCCCTCTCAGAT | CTGGCGGAGGTCAATTCTGT |
|  | *NLRC4* | TCAGAAGGAGACTTGGACGAT | GGAGGCCATTCAGGGTCAG |
|  | *NLRP1* | GCAGTGCTAATGCCCTGGAT | GAGCTTGGTAGAGGAGTGAGG |
|  | *NLRP3* | AGCCACGCTAATGATCGACT | AACCCATCCACTCCTCTTCA |
|  | *NLRP6* | CCTACCAGTTCATCGACCAGA | CTCAGCAGTCCGAAGAGGAA |
|  | *NOTCH1* | GGGTACAAGTGGGACTGTGA | GGTTCAGACATGGGTTGGAC |
|  | *NOTCH2* | ACCCTTGTGAGAATGCTGCT | CCATACCACTGAAGCCTGGT |
|  | *SNAI1* | ATCGGAAGCCTAACTACAGCGA | CACGCCTGGCACTGGTACTTCT |
|  | *YAP1* | TAGCCCTGCGTAGCCAGTTA | TCATGCTTAGTCCACTGTCTGT |
| ***Mouse*** | *Aim2* | GTCCTCAAGCTAAGCCTCAGA | CACCGTGACAACAAGTGGAT |
|  | *Asc* | GACAGTGCAACTGCCAGAAG | CGACTCCAGATAGTAGCTGACAA |
|  | *Casp1* | AGGAATTCTGGAGCTTCAATCAG | TGGAAATGTGCCATCTTCTTT |
|  | *Gsdmd* | ATGCCATCGGCCTTTGAGAAA | AGGCTGTCCACCGGAATGA |
|  | *Hmgb1* | GGGAGGAGCACAAGAAGAAG | TCATAACGAGCCTTGTCAGC |
|  | *Il1β* | GCTTCAGGCAGGCAGTATC | AGGATGGGCTCTTCTTCAAAG |
|  | *Il18* | ACCAAGTTCTCTTCGTTGAC | TCACAGCCAGTCCTCTTAC |
|  | *Nlrc4* | GAAACACTGTACGATCAGCTCC | CATGTTCTTGAAGCGATGGTTTT |
|  | *Nlrp1a* | GGACCTCATGGTGGTTACTTTC | TCCCAGGGGCCGTAAACTT |
|  | *Nlrp1b* | AGTAATCTGGAGGGGTTGGAC | GTTGGCAGCCAGGGTATATCA |
|  | *Nlrp3* | ACCAGCCAGAGTGGAATGAC | ATGGAGATGCGGGAGAGATA |
|  | *Nlrp6* | TCTCTCCGTGTCAGCGTTCA | CGGAAGAGCCGATTAAAAGTGT |
|  | *Tnf* | CAGGCGGTGCCTATGTCTC | CGATCACCCCGAAGTTCAGTAG |

**Supplementary Table 2** Antibodies used for Western Blotting

| **Antibody** | **Company** | **RRID** | **Dilution** | **Primary Antibody Buffer** |
| --- | --- | --- | --- | --- |
| Asc | CST #67824T | AB_2799736 | 1:1000 | 5% BSA / TBS-0.1% Tween |
| BiP (GRP78) | CST #3177P | AB_2119845 | 1:1000 | 5% Skim Milk /TBS-0.1% Tween |
| Erp44 | CST #3798P | AB_1642195 | 1:1000 | 5% Skim Milk /TBS-0.1% Tween |
| Gapdh | CST #2118S | AB_561053 | 1:1000 | 5% Skim Milk /TBS-0.1% Tween |
| Grp94 | CST #2104P | AB_823506 | 1:1000 | 5% Skim Milk /TBS-0.1% Tween |
| HMGB1 | CST #6893 | AB_10827882 | 1:1000 | 5% BSA / TBS-0.1% Tween |
| cleaved Il1β* | CST #63124S | AB_2799639 | 1:1000 | 5% BSA / TBS-0.1% Tween |
| Pro-Il1β | Bioss #bs-6319R | AB_2923270 | 3:2000 | 5% BSA / TBS-0.1% Tween |
| Nlrp3 | CST 15101T | AB_2722591 | 1:1000 | 5% BSA / TBS-0.1% Tween |
| Secondary | CST 7074P2 | NA | 1:1000 | Same as primary |

*used to detect pro-IL1β in mouse placenta; NA, not applicable

**Supplementary Table 3** Antibodies used for immunohistochemistry.

| **Antibody** | **Company** | **RRID** | **Working concentration** |
| --- | --- | --- | --- |
| ***Human*** |  |  |  |
| ASC | CST #13833 | AB_2798325 | 0.14µg/ml |
| Caspase-1 | Invitrogen, #PA5-29342 | AB_2546818 | 2.5 µg/ml |
| cleaved Caspase-1 | Invitrogen #PA5-38099 | AB_2554702 | 2.5 µg/ml |
| GSDMD | CST #96458 | AB_2894914 | 1.0 µg/ml |
| GSDMD^NT^ | CST #36425 | AB_2799099 | 0.9 µg/ml |
| HMGB1 | CST #6893S | AB_10827882 | 1.43 µg/ml |
| cleaved IL1β | Invitrogen #PA5-105048 | AB_2816521 | 2.0 µg/ml |
| ***Mouse*** |  |  |  |
| Caspase-1 | CST #24232 | AB_2890194 | 0.25 µg/ml |
| cleaved Caspase-1 | CST #89332 | AB_2923067 | 0.37 µg/ml |
| GSDMD | CST #39754 | AB_2916333 | 0.47 µg/ml |
| GSDMD^NT^ | CST #10137 | AB_292068 | 0.5 µg/ml |
| HMGB1 | CST #6893S | AB_10827882 | 1.43 µg/ml |
| cleaved IL1β | Invitrogen #PA5-105048 | AB_2816521 | 2.0 µg/ml |

**Supplementary Figures.**


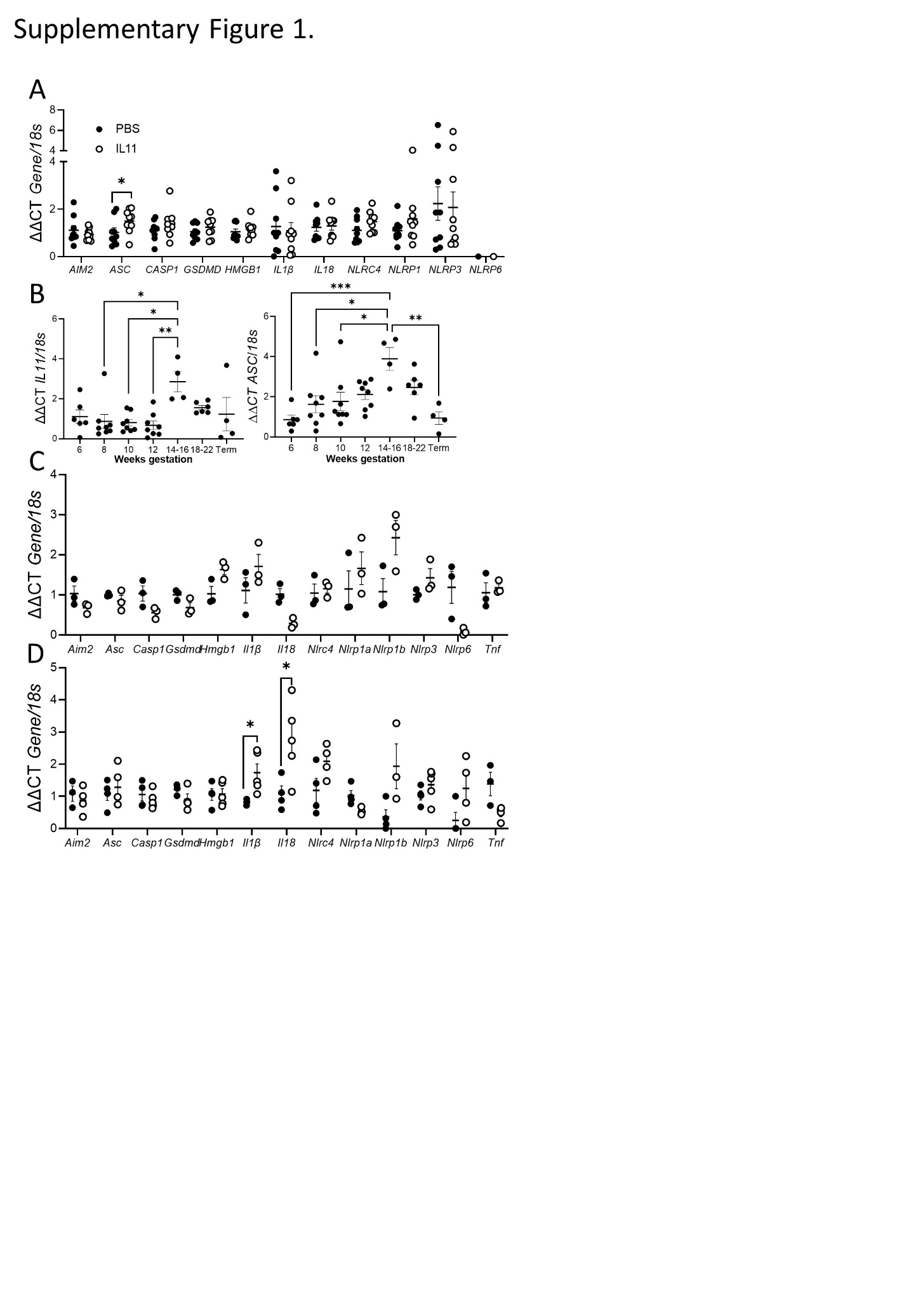


Supplementary Figure 1. A. mRNA expression in human placental villous explants at 22h following IL11 treatment in vitro. B. mRNA expression of *IL11* and *ASC* in human placental villous across gestation. C. mRNA expression in wild-type E13 placenta 2h after IL11 treatment. D. mRNA expression in wild-type E13 placenta after 3 days (E10-E12) of PEGIL11 treatment. ●, PBS/PEG treatment; ○ IL11/PEGIL11 treatment; Data shows mean+SEM; Statistical tests: Wilcoxon matched-pairs signed rank test: A;; One-way ANOVA: B; Mann-Whitney test: C-D; *, p<0.05; **, p<0.01; ***, p<0.001.


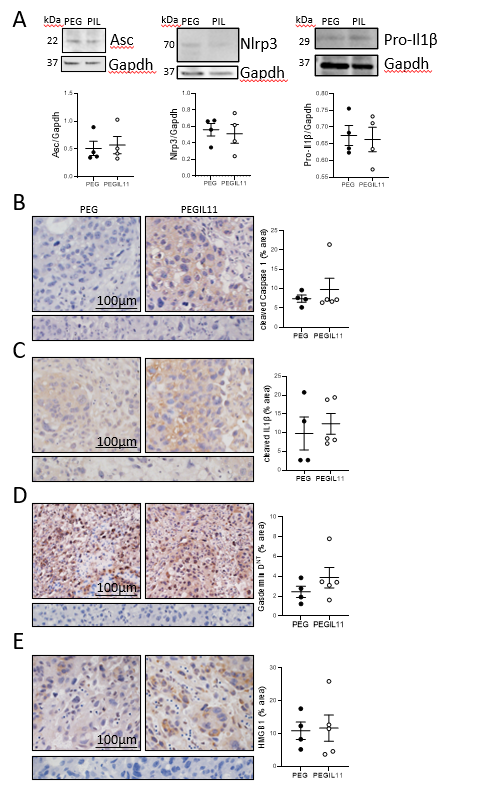


Supplementary Figure 2. Placental protein from E13 wild-type mice treated with PEG/PEGIL11 from E10-12. A. Immunoblots for Asc, Nlrp3 and pro-Il1β with Gapdh as loading control. B-E. Immunostaining of E13 placental labyrinth zone showing PEGIL11 treatment had no effect on (cleaved) caspase-1 (B), IL1β (C), gasdermin D (D; GSDMD^NT^) and increased HMGB1 immunostaining (E) in Asc-/- mice. ●, PEG treatment; ○, PEGIL11 treatment; neg, negative control; PIL, PEGIL11; Data shows mean+SEM; Statistical test: Mann-Whitney test.


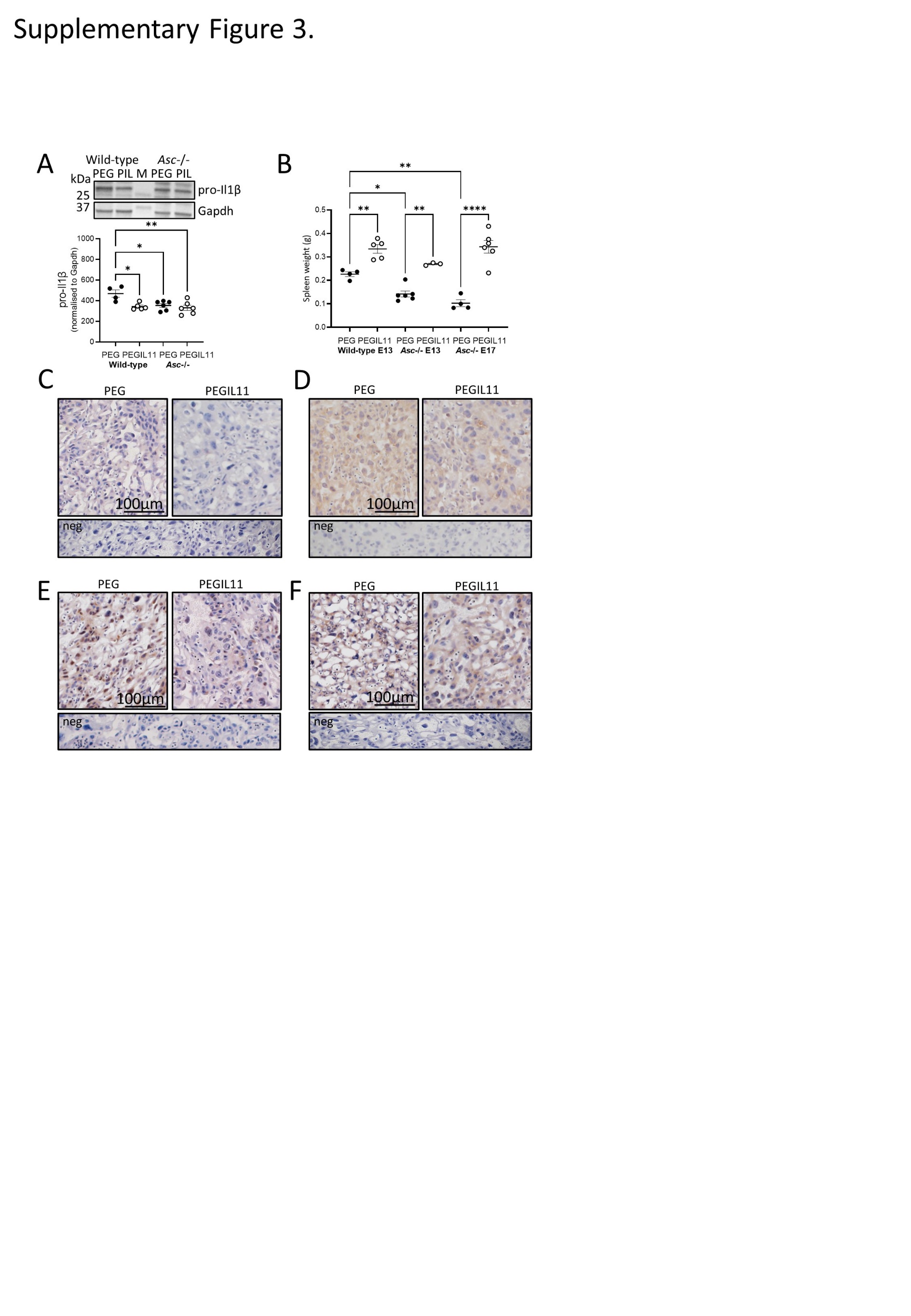


Supplementary Figure 3. A. Immunoblot showing kidney pro-Il1β protein at E13 in wild-type and *Asc*-/- mice after 3 days (E10-12) PEGIL11 treatment. B. Spleen weight at E13/17 in wild-type and Asc-/- mice after 3 days (E10-12; E13 data) or 7 days (E10-E16; E17 data) PEGIL11 treatment. C-F. Immunostaining of E13 placental labyrinth zone showing PEGIL11 treatment had no effect on activated (cleaved) caspase-1 (B), IL1β (C), gasdermin D (D; GSDMD^NT^) and HMGB1 immunostaining (E) in *Asc*-/- mice (n=4-5/group). ●, PEG treatment; ○ PEGIL11 treatment; neg, negative control; PIL, PEGIL11; Data shows mean+SEM; Statistical tests: One-way ANOVA: A,B; *, p<0.05; ** p<0.01; ***, p<0.001; ****, p<0.0001.


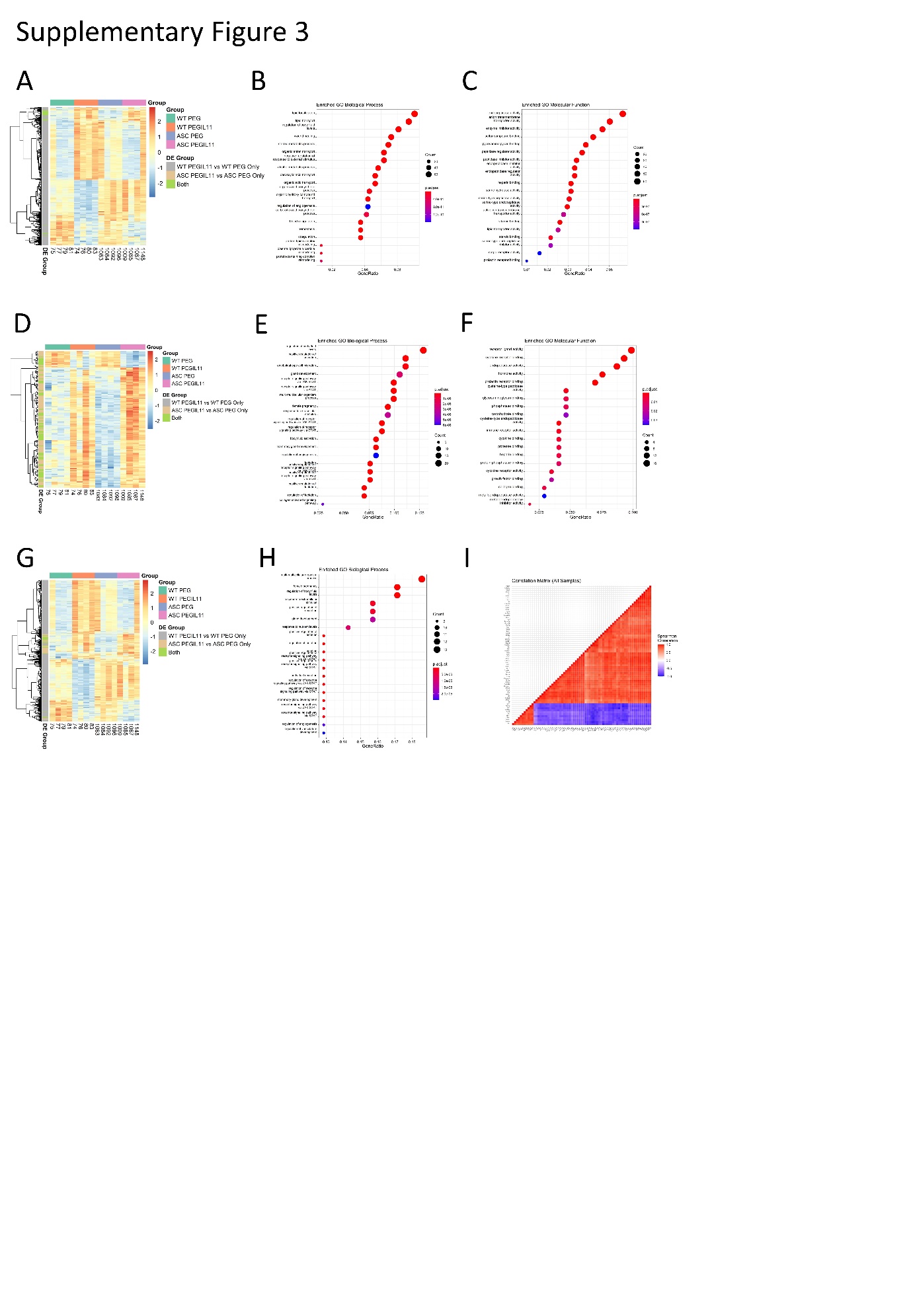


Supplementary Figure 4. RNAseq of placenta from wild-type and *Asc*-/- placenta following treatment with PEG and PEGIL11. A-C. Wild-type placenta following PEG vs PEGIL11 treatment: A. Heatmap of differentially expressed genes; B. Enriched GO Biological Process; C. Enriched GO Molecular Function. D-F. *Asc*-/- placenta following PEG vs PEGIL11 treatment: D. Heatmap of differentially expressed genes; E. Enriched GO Biological Process; F. Enriched GO Molecular Function. G-I. Differentially expressed genes found in both wild-type and *Asc*-/- placenta following treatment with PEGIL11: G. Heatmap of differentially expressed genes; H. Enriched GO Biological Process; I. Correlation of differentially expressed genes in both wild-type and *Asc*-/- placenta following treatment with PEGIL11.


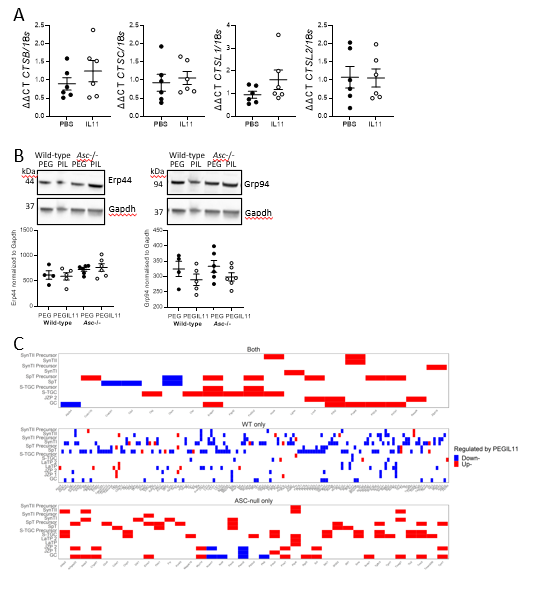


Supplementary Figure 5. A. Cathepsin mRNA expression in human placental villous explants treated with IL11 (100ng/ml) for 22h. B. Immunoblot showing E13 placental ER molecular chaperone protein expression: Erp44, Grp94 normalized to Gapdh E13 in wild-type and *Asc*-/- mice treated with PEGIL11 (500µg/kg/day) from E10-E13. C. Regulated trophoblast cell linage markers by PEGIL11 treatment in wild-type and *Asc-/-* placenta. ●, PBS/PEG treatment; ○, IL11/PEGIL11 treatment; PIL, PEGIL11; Data shows mean+SEM; Statistical tests: Wilcoxon matched-pairs signed rank test, A; One-way ANOVA, B.


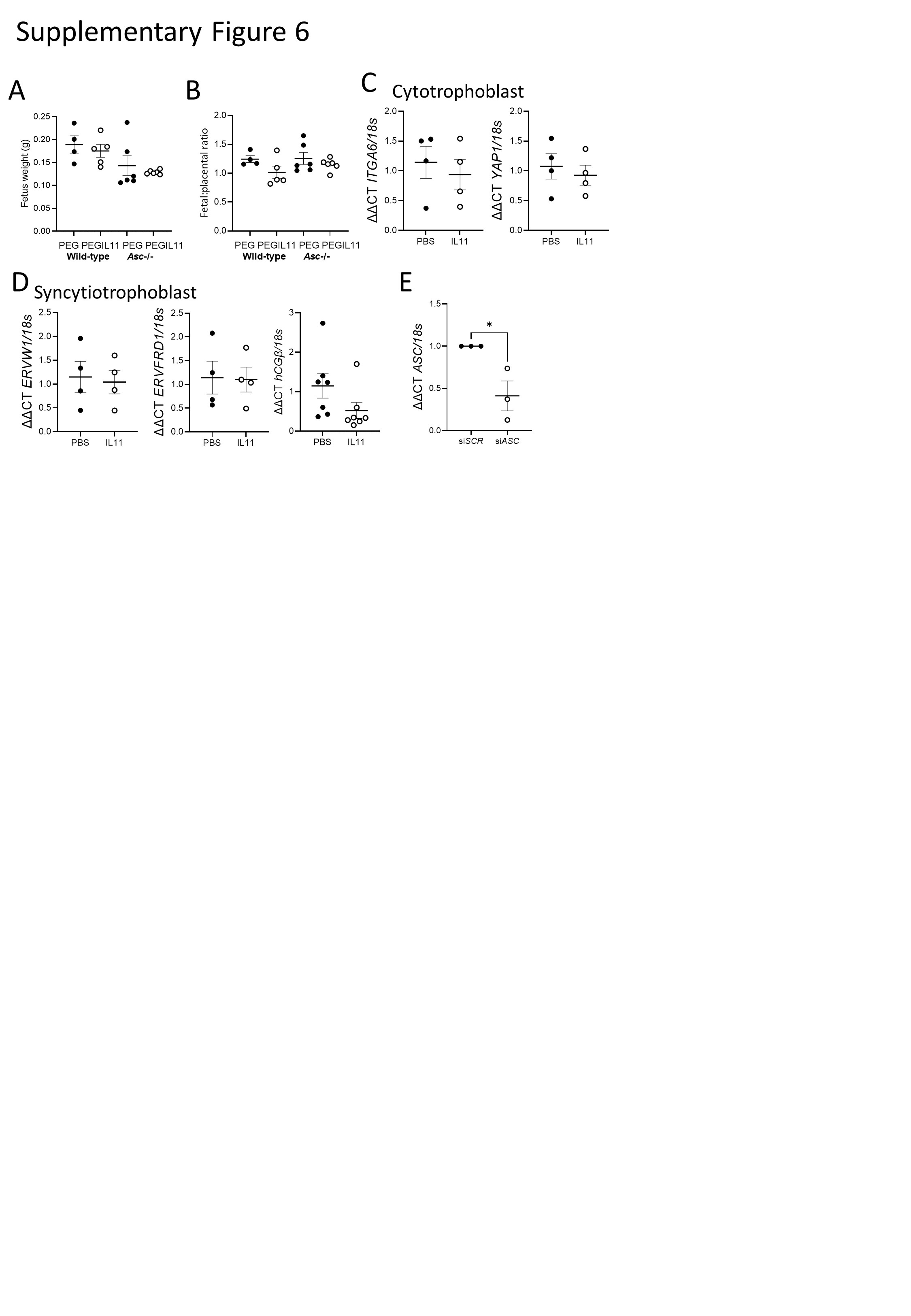


Supplementary Figure 6. A. Fetal weight at E13 in wild-type and *Asc*-/- mice treated with PEGIL11 (500µg/kg/day) from E10-E13. B. Fetal:Placental ratio at E13 in wild-type and *Asc*-/- mice treated with PEGIL11 (500µg/kg/day) from E10-E13. C-E. Markers of trophoblast linage mRNA expression in human placental villous explants treated with IL11 (100ng/ml) for 22h. D. cytotrophoblast, E. syncytiotrophoblast, E. Confirmation of *ASC* knockdown in villous tips by qPCR. ●, PEG/PBS treatment; ○, PEGIL11/IL11 treatment; Data shows mean+SEM; Statistical tests: One-way ANOVA:, A,B; Wilcoxon matched-pairs signed rank test: C-D; Paired t-test E
